# Disruptions in retinoic acid signaling pathway contribute to abnormal lung development in congenital diaphragmatic hernia: a therapeutic potential for retinoids to attenuate pulmonary hypoplasia

**DOI:** 10.1038/s41390-024-03086-7

**Published:** 2024-02-10

**Authors:** Florian Friedmacher, Prem Puri

**Affiliations:** 1 Department of Pediatric Surgery, University Hospital Frankfurt, Goethe University Frankfurt, Frankfurt, Germany; 2 Beacon Hospital, University College Dublin, Dublin, Ireland

The development of the diaphragm has been a source of fascination for scientists and clinicians for centuries. With an estimated global prevalence ranging between 2.3 and 2.6 per 10,000 births,^1,2^ congenital diaphragmatic hernia (CDH) is a relatively common birth defect, characterized by incomplete formation and/or muscularization of the diaphragm. Consequently, abdominal organs herniate through the diaphragmatic defect into the ipsilateral thoracic cavity, thereby occupying space normally reserved to accommodate the growing lung. Thus, pulmonary development is often severely disturbed in fetuses with CDH, leading almost invariably to immature and hypoplastic lungs.^3^ Depending on the degree of pulmonary hypoplasia (PH), most newborns with CDH present with life-threatening respiratory distress at birth, requiring immediate and complex treatment. Although significant improvements have been achieved in postnatal resuscitation and ventilation strategies over the past decades, CDH still represents one of the major challenges in neonatal intensive care with mortality rates ranging between 30% and 50%.^4,5^ While from a surgical perspective it is relatively easy to repair the diaphragmatic defect either by primary closure or reconstruction using a prosthetic patch or muscle flap, lung growth is already critically disrupted by this time point. In fact, mortality and long-term morbidity in CDH are directly related to the severity of PH. Most of our current knowledge about the pathogenesis of CDH and associated morphological changes in hypoplastic lungs has originated from experimental animal models.^6^ Nevertheless, the molecular and cellular mechanisms underlying CDH and PH remain poorly understood.

In their recent review article published in *Pediatric Research*, Rivas and Clugston^7^ present a comprehensive update on the “*retinoid hypothesis*”, drawing upon scientific work from animal models, human genetics and epidemiological studies of CDH published since the original landmark paper by Greer et al.^8^ from 2003. The authors provide strong evidence, which links defective retinoic acid (RA) signaling to the pathogenesis of CDH, mainly focusing on the RA signaling pathway in abnormal diaphragm development.^7,8^ However, while the diaphragm is undoubtedly central in the etiology of CDH, most of the current research efforts have been concentrating on the pathogenesis and treatment of associated PH.^9^ Typical features of hypoplastic lungs in CDH are structural immaturity and smaller size with a significantly reduced number of terminal airway generations, disrupted alveologenesis, diminished alveolar airspaces, thickened alveolar walls accompanied by increased interstitial tissue and decreased gas-exchange surface area.^10^ Although PH was initially discussed as a secondary defect in CDH due to abdominal organ herniation via physical compression, the “*dual-hit hypothesis*” suggested a primary disruption in bilateral lung organogenesis before closure of the diaphragm combined with a second ipsilateral insult resulting from intrathoracic herniation and subsequent restriction of fetal breathing movements.^11^ This hypothesis is further supported by human cases of CDH, where only minimal organ displacement is observed, yet PH still occurs.^12^

Despite starting from different pathways and anlages, today it is well-established that diaphragm and lung development are interrelated. RA, one of the most biologically active metabolites of vitamin A (i.e. retinol) and its derivates (i.e. retinoids), are essential components of the complex gene network that regulates formation of the diaphragm and lung morphogenesis.^13,14^ During fetal lung development, RA and retinoids are crucial for each of the five developmental stages.^15–17^ There is a chronic vitamin A deficiency among women of child-bearing age with an increased demand for vitamin A during this pivotal period of gestation.^18^ Moreover, transcriptional analysis of key components of RA signaling in human and animal CDH lungs confirmed disruptions in the retinoid pathway as part of CDH pathogenesis,^19,20^ which was associated with reduced pulmonary RA and retinol levels.^21,22^ For that reason, prenatal administration of RA is being widely studied as a therapeutic tool to improve lung maturation in preclinical models of CDH.^23,24^ For instance, it was recently reported that in vitro treatment with RA stimulates avian lung branching through a complex retinoid network.^25^ Additionally, RA is known to be critically involved in the saccular phase through stimulation of alveolar epithelial cell type I and II proliferation.^26,27^ A previous study reported a 50% increase in the number of pulmonary alveoli in newborn rats after treatment with RA, suggesting an important role of retinoids during alveolarization.^28^ Findings from animal experiments revealed that disruption of RA signaling contributes to the formation of CDH and hypoplastic lungs.^29^ Furthermore, it was demonstrated that prenatal administration of RA during late gestation upregulates pulmonary expression of several genes involved in the retinoid signaling pathway.^30^ It was also shown that RA reduces the severity of PH in nitrofen (an herbicide)-induced hypoplastic lung explants (in which retinal dehydrogenase 2 is inhibited)^31^ and rescues PH in calorie-restricted developing rat lungs.^32^ Additional evidence that prenatal administration of RA stimulates alveolarization in hypoplastic lungs was provided by in vivo studies in rats with nitrofen-induced CDH,^33–37^ indicating that RA may have a therapeutic potential in attenuating CDH-associated PH. In surgical models of CDH, prenatal administration of RA normalized alveolar epithelial cell differentiation in rabbits,^24^ whereas treatment with vitamin A improved lung morphology and function in lambs.^38^ The use of retinoids during pregnancy remains controversial and is currently restricted by the Food and Drug Administration because of its teratogenic side effects.^39^ Yet, pregnant women with acute leukemia were successfully treated with RA during the second and third trimester of pregnancy with no adverse effects on the newborn.^40,41^ This would allow a theoretical time window for the safe administration of retinoids during late gestation when alveolarization of fetal lungs begins. Therefore, in future it might be possible to intervene prenatally with vitamin A and RA to reduce PH, lessening the impact of CDH. However, further research is needed to establish the exact molecular and cellular effects of retinoid treatment on fetal lung development in CDH. Accordingly, international collaborations and translational research should be strengthened to improve outcome of CDH patients in the 21st century.^42^
